# Transfer in Rule-Based Category Learning Depends on the Training Task

**DOI:** 10.1371/journal.pone.0165260

**Published:** 2016-10-20

**Authors:** Florian Kattner, Christopher R. Cox, C. Shawn Green

**Affiliations:** 1 Department of Psychology, University of Wisconsin-Madison, Madison, WI, United States of America; 2 Department of Psychology, Technische Universität Darmstadt, Darmstadt, Germany; Waseda University, JAPAN

## Abstract

While learning is often highly specific to the exact stimuli and tasks used during training, there are cases where training results in learning that generalizes more broadly. It has been previously argued that the degree of specificity can be predicted based upon the learning solution(s) dictated by the particular demands of the training task. Here we applied this logic in the domain of rule-based categorization learning. Participants were presented with stimuli corresponding to four different categories and were asked to perform either a category discrimination task (which permits learning specific rule to discriminate two categories) or a category identification task (which does not permit learning a specific discrimination rule). In a subsequent transfer stage, all participants were asked to discriminate stimuli belonging to two of the categories which they had seen, but had never directly discriminated before (i.e., this particular discrimination was omitted from training). As predicted, learning in the category-discrimination tasks tended to be specific, while the category-identification task produced learning that transferred to the transfer discrimination task. These results suggest that the discrimination and identification tasks fostered the acquisition of different category representations which were more or less generalizable.

## Introduction

With practice, humans tend to improve their performance on most tasks. However, the performance enhancements that arise through practice are often annulled if the task or stimulus is changed. This is sometimes referred to as the ‘curse of specificity’ [1,2], and it has been observed in some form or another in virtually every sub-domain where learning has been examined—from education [3], to motor control [4], to cognition [5,6].

While the curse of specificity is a general phenomenon, it has been perhaps most clearly observed and extensively studied in the domain of perceptual learning [7–9]. For example, consider the classic vernier acuity task. Participants are presented with two vertical lines, one above the other, with the top line displaced to the left or right of the bottom line. The task is to indicate the direction of the displacement. Repeated practice with this task results in clear enhancements in task performance. Critically though, if participants who have been trained to detect the offset of vertical lines are then asked to detect the offset of horizontal lines (i.e., the display is rotated 90 degrees), performance returns to baseline. In other words, none of the benefits of training on the vertical version of the task transfer to the horizontal version of the task (or vice versa; [10,11]. In all, this kind of task-specific learning has been observed for myriad low-level features and training characteristics including retinal position [12], spatial frequency [13], motion direction [14], motion speed [15], and even the trained eye [16].

Yet, while specificity of learning has been perhaps the most common finding in the field to date, emerging research has suggested that significant generalization is possible in some cases. What distinguishes those studies that have observed learning generalization from their more classical counterparts is the use of training paradigms that incorporate a variety of characteristics which have each been independently shown to encourage learning and transfer [17]. Such characteristics include training on more complex tasks (e.g., “real-world” activities–such as playing music, [18], or video games, [19]), training with multiple tasks (e.g., [20–23], utilizing a variable set of stimuli (in terms of spatial frequency, orientation, and spatial location), ensuring a proper level of difficulty (e.g., challenging, but doable), and/or providing external rewards to maximize motivation and engagement (e.g., [1,24]).

The work cited above, as well as that by many others, has led to the argument that the specificity or generality of learning is dictated by task demands rather than stimulus properties [10,25–27]. Under this viewpoint, participants will tend to learn the simplest and least error prone solution to a given task. Thus, by knowing the simplest solution to a learning problem, one can predict the range of tasks and circumstances that a training paradigm will impact.

Given such a prediction, it is possible also to design paradigms to encourage more or less specificity of learning. In general, more transfer is expected with learning solutions that involve the acquisition of abstract rules or predictive models as compared to learning solutions that involve simple boundaries or other types of “model-free” stimulus-response mappings. For example, in orientation discrimination learning, Green and colleagues contrasted two different training tasks [27]. In both tasks, on each trial, participants were presented with a Gabor patch whose orientation was drawn from a uniform distribution from 25° to 65°. One group of participants performed a simple categorization task, in which they were asked to indicate whether the given Gabor was oriented clockwise or counter-clockwise relative to 45°. The second group was also asked to make a clockwise/counterclockwise judgement on each trial. However, the standard against which they were asked to judge changed on every trial (e.g., on one trial they may have been asked to indicate if the Gabor was clockwise or counterclockwise relative to 35° and on the next trial they may have been asked if it was clockwise or counterclockwise relative to 50°). In the first group, the task can be easily solved by learning a boundary that separates the space of Gabors into ‘clockwise from 45°’ and ‘counter-clockwise from 45°’ categories. Crucially, this boundary is then of no use in a transfer task where stimuli and reference angle is changed (e.g., to discriminations around 135°). In the second group, it is not possible to learn a simple boundary since the category boundaries are new on every trial. It is thus necessary to learn to more flexibly determine the orientation of a presented Gabor relative to an ever-changing standard. Clearly, this ability is then still useful in a transfer task where stimuli and reference angle are changed. And indeed, this is what Green et al. [27] observed. Despite the groups having nearly identical visual experience during training, the difference in the tasks that the groups were asked to perform resulted in very different generalization outcomes. As predicted, significant specificity was observed in the group that learned to categorize stimuli relative to a constant reference angle, while transfer was observed in the group trained with a continuously changing reference angle.

Because the general framework described should be relatively domain independent (i.e., it should not be only applicable to perceptual training), the present work is an attempt to assess these predictions in a more cognitive category learning paradigm. That different training tasks produce different learning outcomes has been long appreciated by those who study categorization at the conceptual level (e.g., when learning about within- and between-category structure among a set of stimuli; [28]; or when learning categories that are separable along a single dimension or multiple dimensions [29,30]). Indeed, accounting for task effects is a basic requirement of any viable model of how categories are learned and used (e.g., [31–33]). For example, a number of studies by Logan and colleagues [34–36] have demonstrated that the encoding and retrieval of information from previous learning episodes depend on where attention was directed during the learning task (e.g., focused versus divided attention during automatization training). There is also evidence that unidimensional discrimination tasks that can be solved by learning an easily stated declarative rule are learned in a fundamentally different way than tasks that require integrating information over multiple dimensions and that this in turn results in differences in terms of the generalization of that learning [29,37–39]. It was found that when a task can be solved with a rule-based strategy, performance will generalize to novel stimuli that respect the rule, while tasks that require information integration do not tend to support generalization [29,40].

This suggests that the task a participant is asked to perform influences what information is attended (e.g., a subset of task-relevant features or a broader range of stimulus features), what is learned (e.g., task-specific or general solutions), and whether the transfer to new stimuli or tasks may be expected. In this same vein, a basic distinction is typically made in the categorization literature between tasks that present participants with exemplars that must be classified into one of several categories (*classification tasks*) and tasks that present participants with a category label and an incomplete image of an exemplar, which must be completed by inferring the missing feature (*inference tasks*; see [41]). Classification tasks require participants to focus on the differences between categories, while inference tasks require knowledge of the relationships among features within categories. As such, training with one task or the other is expected to lead to different patterns of transfer performance. Specifically, more transfer would generally be expected for inference learners who acquire information about the internal structure of a category (covering all features that define a prototype of the category) than for classification learners who focus on specific features that differentiate categories.

Consistent with this viewpoint, it was shown that typicality ratings after classification learning were affected only by features that differentiated the two categories, whereas typicality ratings after inference learning were influenced by any (prototypical) features regardless of whether they were relevant for category discrimination [42]. This pattern of results indicates that classification learners give more weight to discriminatory features, while all prototypical features are weighted equally by inference learners [43]. It was also found that within-category feature co-occurrences, category coherences, as well as queried features (as opposed to diagnostic/discriminatory features) are learned and memorized better with inference tasks than with classification tasks [44–46].

It is thus not surprising that training on inference tasks has been found to transfer to classification tasks, and even permits better classification of novel stimuli than classification training [47]. Conversely, classification training does not transfer well to inference tasks. For instance, while classification learners typically perform better on classification tests in which the full stimuli were presented, it was also found that inference learners performed better on classification tests with single features, suggesting that inference tasks leads to learning about all features of the category [48]. This general pattern thus maps nicely onto the theoretical framework and empirical results seen in the perceptual learning domain described above–particularly in terms of the differences in transfer that can be expected from learning a generative model versus learning a model-free solution.

The goal of the present work was thus to further explore the extent to which the task performed during learning alters the amount of learning generalization that is observed. More specifically, as was done in recent work in the perceptual learning domain (e.g., [10,27]), our objective here in the categorization domain was to design multiple rule-based category learning tasks that were matched for visual experience, but which required different learning solutions. These learning solutions were then predicted to result in different patterns of generalization.

**Fig 1:**
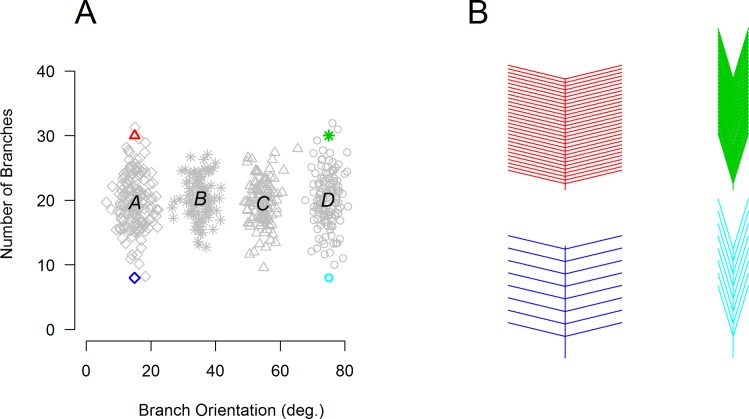
Categories of feathers as defined by the number and orientation of branches in an example subject. Panel A illustrates the 500 feathers resulting from 2D Gaussian distributions which were presented to a participant during training. Category membership is indicated by unique symbols (diamonds, stars, triangles, and circles). Panel B illustrates 4 example feathers drawn from these distributions. Note that the colored symbols in panel A refer to the feathers in the corresponding colors in panel B.

**Fig 2:**
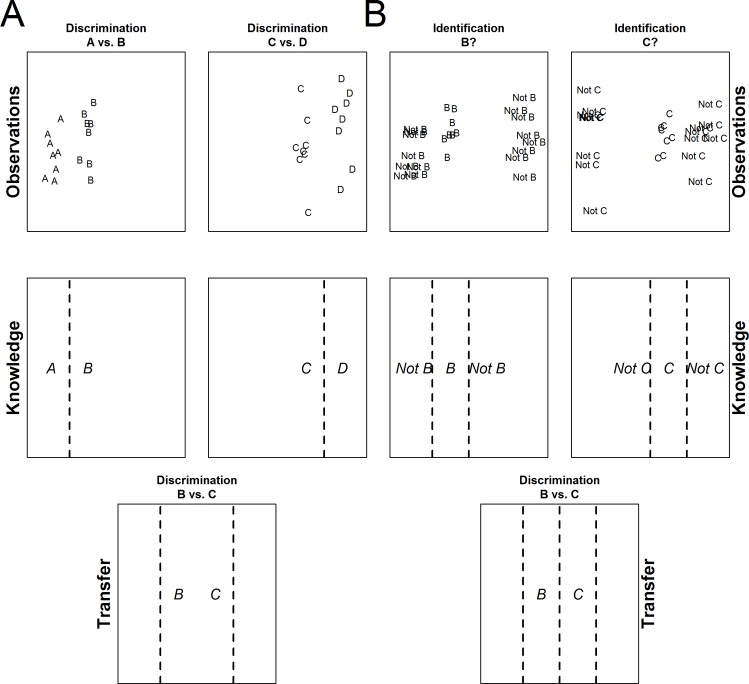
**Illustration of the most likely learning solutions in a 2-alternative category discrimination task (panel A) and in a category identification task (panel B).** In a 2-alternative discrimination task, participants are presented with feedback on whether the observations belong to one of *two* possible categories (corresponding to the response options; e.g., ‘A vs. B’), and they are likely to learn rules, i.e., the discriminatory boundaries between two distributions. This knowledge, however, is insufficient to immediately discriminate the B and C distributions (i.e., no transfer is expected). In contrast, in an identification task, participants are given feedback on whether the observations belong to *one* particular category or not, and participants are likely to learn two-sided category boundaries, and should subsequently be able to immediately solve the B/C discriminations (i.e., transfer is expected).

To this end, we created a simple continuous 2D stimulus space (see Fig 1) from which novel stimuli could be drawn (cf. [30]). This space was organized into four categories that could be discriminated along a single dimension (for exposition purposes below, we refer to these as Categories ‘A’, ‘B’, ‘C’, and ‘D’). Participants in all training task conditions were exposed to the same basic stimuli drawn from all four of these categories, thus ensuring that any differences in transfer effects can be interpreted in terms of task demands rather than in terms of simple experience with certain stimuli; see methods below. However, despite utilizing the same stimuli, the various training tasks were designed with the goal of either generalizing to, or failing to generalize to, a final transfer discrimination task that was identical across all groups. In this final transfer task, all participants were asked to categorize stimuli as belonging to one of the two ‘middle’ distributions (i.e., Categories ‘B’ and ‘C’) in the 2D stimulus space (see Fig 2). Therefore, when constructing our training tasks, we carefully considered the types of training that should or should not promote generalization to this particular transfer task.

First, and critically, none of the training tasks explicitly contained this exact categorization (i.e., although participants saw many examples of stimuli from both Category ‘B’ and Category ‘C’ during training, at no point during training in any task were any participants directly asked the question of whether a given stimulus belonged to ‘Category B or Category C’). Next, as discussed above, tasks that can be solved via learning specific discrimination rule (e.g., a discriminatory boundary on a particular dimension) tend not to produce transfer to tasks that require a new rule (e.g., an untrained category discriminant). As such, three of the training tasks shared the general property that asymptotic performance could be achieved by learning a series of unidimensional discrimination rules (though they differed slightly in other ways that previous research suggested might alter the extent to which generalization was observed). Because the discrimination between the ‘B’ and ‘C’ distributions was always left out of these training tasks though, our expectation was that significant specificity would be observed when participants were asked to make this particular discrimination in the transfer task (see Fig 2). Conversely, the fourth task (i.e., the “identification task”) involved feedback that did not easily allow for a simple rule to be learned (see below and Fig 2). Instead, the simplest solution for this task would be to learn a ‘band’ around the various categories. Because learning these bands would in turn allow one to perform the ‘B versus C’ discrimination task (i.e., if one learned to assess whether a given stimulus was within the ‘B’ band or the ‘C’ band, then one could obviously indicate whether the same stimulus was a ‘B’ or a ‘C’), significant transfer of learning was expected after training with the identification task.

It is important to highlight the fact that, although this general approach and the specific experimental predictions were derived from work in both the perceptual learning and categorization literatures, the implementation is somewhat unique. In particular, the most common methodology in both of those respective domains involves assessing transfer to new categories or stimuli (e.g., “Does a rule that was learned while training on one set of stimuli transfer to a previously unseen set of stimuli that can be separated by the same rule?”). In our case, the stimuli utilized during the transfer test were not new to the participants–indeed, the participants had not only observed the transfer stimuli throughout training, they had to make a variety of judgements about the category membership of those stimuli. The critical question was instead whether the training allowed the participants to make an untrained decision about those previously observed stimuli.

## Materials and Methods

### Ethics statement

This research was approved by the University of Wisconsin-Madison Education and Social/Behavioral Sciences Institutional Review Board. All participants provided written informed consent prior to participation.

### Participants

A total of 71 undergraduate students were recruited at the campus of the University of Wisconsin-Madison. Each participant was assigned to one of four different training task conditions: *Blocked Discrimination Training* (*n* = 28; 18 females; age: 18–24 years; *M*_*age*_ = 19.4; *SD*_*age*_ = 1.5), *Intermixed Discrimination Training* (*n* = 11; 7 females; age: 18–21 years; *M*_*age*_ = 19.5; *SD*_*age*_ = 0.9), *Triplet Discrimination Training* (*n* = 14; 8 females, age: 18–20 years; *M*_*age*_ = 18.9; *SD*_*age*_ = 0.9), or *Identification Training* (*n* = 18; 10 females; age:18–21 years; *M*_*age*_ = 19.4; *SD*_*age*_ = 0.9). We note that because this was a new task, more participants were planned in the blocked condition (goal *N* = 30) as this was both the simplest possible learning condition and the condition where the greatest specificity was predicted). The larger *N* would confirm that A) the task was learnable and B) that there was room to observe differences in transfer in other training conditions. For the remaining conditions, our target goal for each condition was *N* = 12, however, the exact cutoff dates depended partially on natural stop points in the semester resulting in slightly different *N*s across the conditions. All participants were compensated with course credit.

### Apparatus and Stimuli

The experimental routines were programmed in MATLAB utilizing the Psychophysics Toolbox extensions [49,50] on a Dell XPS computer running Windows 7. Stimuli were presented by an NVIDIA GeForce 8800 GTX video card on a 22-inch wide-screen Dell LCD monitor with a resolution of 1680 x 1050 pixels. Subjects were seated approximately 60 cm from the screen.

The stimuli were simple white line drawings (schematic feathers) displayed on a gray background (see Fig 1). Each drawing consisted of a central vertical line of random length and a variable number of lines branching off symmetrically to the left and the right. The lengths of the branches differed and were determined randomly on each trial. The four different categories of feathers were defined by multivariate normal distributions of the orientation and the number (density) of branches, with only one dimension being relevant for the discrimination of categories in both learning phases. For twelve participants trained on the *Blocked Discrimination* task (7 female), the relevant dimension was the number of branches on each side of the vertical (μ_A_ = 6; μ_B_ = 18; μ_C_ = 30; μ_D_ = 42; σ = 4 lines), and the orientation was drawn from a normal distribution with μ = 40° and σ = 9°. For the remaining sixteen participants that were trained on this task (11 female), as well as for participants trained on any of the other tasks, the relevant dimension was the orientation of the branches (μ_A_ = 15°; μ_B_ = 35°; μ_C_ = 55°; μ_D_ = 75°; σ = 3°), and the density was defined by μ = 20 and σ = 4 lines on each side.

### Procedure

#### Overall procedure

The basic experimental design was identical for all groups (see Table 1). On each trial, a single feather drawing (with the number and orientation of the branches being drawn from the respective 2D normal distribution) was presented. In all groups, the training phase required participants to perform some task (see below) involving all four categories—A, B, C, and D (note that the means of the normal distributions defining the categories are ordered along the relevant dimension with A<B<C<D). Crucially though, the B/C discrimination was never presented during the training stage in any group. The training was followed by a transfer phase in which participants were required to learn the crucial B/C discrimination (see Table 1 for details). Although we use the labels A, B, C, and D to describe these categories for ease of exposition, note that in the actual experiment, the categories were given arbitrary non-word category labels. This insured that participants could not infer any information about the categories or their ordering from the label names. For each participant, four different labels were drawn randomly from a set of 16 non-words (*Alic*, *Bork*, *Cosa*, *Dala*, *Elpo*, *Furn*, *Gort*, *Hust*, *Iola*, *Joca*, *Knos*, *Liaw*, *Mamp*, *Nuse*, *Olpe*, *Pald*).

**Table 1:** Experimental design of the two category learning phases for the different groups: In the Blocked and Intermixed Discrimination tasks, stimuli were categorized with regard to one of two category labels on each trial. In the Triplet Discrimination task, stimuli were to be assigned to one of either two or three category labels on each trial. In the Identification task, only one category label was presented (the category in parenthesis was not shown), and participants had to decide whether the stimulus belonged to that category or not. The transfer phase was the same for all groups. Stimuli had to be assigned to one of two category labels (B and C) which were never explicitly discriminated before (but all groups separately classified instances of both B and C).

	Blocked Discrimination	Intermixed Discrimination	Triplet Discrimination	Identification
**Training**	50-trial blocks:	500 trials mixed:	504 trials mixed:	500 trials mixed:
	2× A/B	100× A/B	72× A/B	50× A _(/B)_
	2× A/C	100× A/C	72× A/C	50× A _(/C)_
	2× A/D	100× A/D	72× A/D	50× A _(/D)_
	2× B/D	100× B/D	72× B/D	50× _(A/)_ B
	2× C/D	100× C/D	72× C/D	50× B _(/D)_
			72× A/B/D	50× _(A/)_ C
			72× A/C/D	50×C _(/D)_
				50× _(A/)_ D
				50× _(B/)_ D
				50× _(C/)_ D
**Transfer**	100× B/C	100× B/C	100× B/C	100× B/C

#### Different training tasks

Blocked Discrimination Training. Ten 50-trial blocks with A/B, A/C, A/D, B/D, and C/D discriminations were presented during the training phase (each of the discrimination problems was presented twice), resulting in a total of 500 trials of training. The order of the ten blocks was counterbalanced across participants. On each trial, a random member of one of the two categories for the given block was presented along with the corresponding two category labels (i.e., either A and B, or C and D). The two category labels were presented as the two ends of a scale. Participants were asked to categorize the presented stimulus by clicking on the scale closer to one end of the scale depending on how confident they were about the particular categorization (i.e., they should have started clicking on the middle of the scale and become more confident across a series of trials). Feedback was presented immediately after the response for 2 seconds, with the feedback indicating the correct category label (e.g., ‘Correct. It was a Dala.’ or ‘Incorrect. It was a Gort.’). Given that participants could, in effect, treat this training phase as consisting of a series of smaller learning tasks–each of which could be solved by learning a specific rule (e.g., the unidimensional boundary dividing categories A and B). Although some of the discrimination boundaries cross the B/C divide (i.e., A/C, A/D, and B/D), we expected only little transfer to the explicit B/C discrimination problem (see Fig 2A).

Intermixed Discrimination Training. 50 trials of each A/B, A/C, A/D, B/D, and C/D discriminations were presented in a fully intermixed order. Apart from that, the procedure was identical to the *Blocked Discrimination Training*. Because the stimuli were otherwise identical between the *Blocked Discrimination Training* and the *Intermixed Discrimination Training* this allowed us to test the effect of interleaving material, which, in some domains has been suggested to increase the generality of learning [51–53].

Triplet Discrimination Training. In this task, either two or three category labels were presented on a trial (i.e., there were trials with pairs and trials with triplets, see Table 1). During the training stage, 72 trials of A/B, A/C, A/D, B/D, C/D, A/B/D, and A/C/D discriminations were presented, resulting in a total of 504 trials (note that fewer repetitions per discrimination were presented in this group to approximately match the total number of training trials in other groups). Here participants had to click the line (either two or three scales were presented on the screen) that was associated with their guess for the category of the presented stimulus with the additional instruction to click farther right the more confident they felt. Feedback was provided in the same manner as in the *Blocked and Intermixed Discrimination Tasks*. To some extent, the addition of the triplets was not predicted to result in any greater degree of generalization. Instead, this condition was mainly meant to serve as a control against which the next condition (*Identification Training*) could be assessed.

Identification Training. In this task, the participants were presented with the exact same stimuli as the *Intermixed Discrimination Training*, but were required to provide a different type of response. However, only one of the two category labels was presented on each trial. The task was thus not a categorization, but was instead an identification. Participants had to indicate whether the presented label corresponded to the shown category or not by pressing the (Y) or (N) key, respectively. Critically, the feedback in this condition only provided information about the validity of the response (e.g., ‘Correct. It was a Liaw.’ or ‘Incorrect. It was not a Liaw.’). Because the stimuli were identical in the *Intermixed Discrimination Training* and the *Identification Training* conditions, but the responses/tasks differed, comparing the behavioral results between these two conditions allows for a clear test of the role of training task uncontaminated by differences in stimuli. We hypothesized that in the *Identification Training* condition participants should be unable to learn simple rules to discriminate the categories on a single dimension. Indeed, as is clear in Fig 2, there is no single category boundary that can separate the types of feedback the participants receive (e.g., there will be stimuli labeled as ‘Not B’s’ on both sides of the stimuli labeled as ‘B’s’ and thus no single line can separate ‘B’ from ‘Not B’). Instead, the best that can be learned here is a ‘band’ separating each category from the items that are ‘not’ that category. Because these bands are then useful for learning the B/C discrimination rule (i.e., if you know if a stimulus is or is not in the ‘B’ band AND if a stimulus is or is not in the ‘C’ band, then you also know whether the stimulus is a ‘B or a C’), we expected to see largely full transfer from training on this task.

#### Transfer task

The transfer task was identical for all four groups. Participants were presented with 100 (i.e., two blocks) discrimination trials with only members of the two categories B and C in random order. The stimuli were to be categorized using the same type of confidence scale as in the *Blocked and Intermixed Discrimination* trainings (i.e., the same labels were presented at the two ends of the scale throughout the transfer stage). Feedback was provided in the same way as during the discrimination training tasks by presenting the true category label together with the word ‘Correct’ or ‘Incorrect’.

## Results

**Fig 3:**
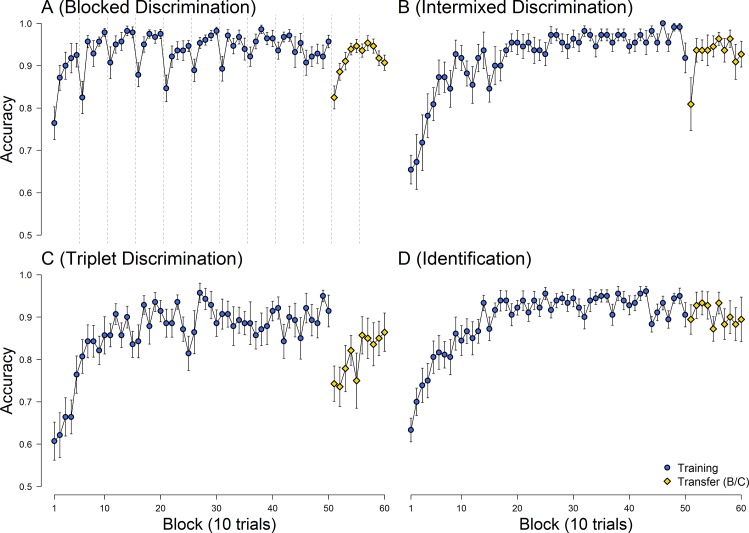
Mean accuracy of categorizations in each 10-trial block during the training and transfer stages in the four ‘feather’ categorization tasks. While no transfer to a previously ‘unseen’ B vs. C category discrimination problem was found with the three types of discrimination tasks (A, B, and C), nearly full transfer to the B vs. C discrimination problem was found when participants were trained on a category identification task (D).

Fig 3 illustrates the average accuracy of categorization responses in each 10-trial block of the training and transfer stages in the four groups who were trained on different categorization tasks with the same stimuli. It is evident that participants successfully learned to categorize the feathers with all training tasks, reaching an average of 93.9% (*SD* = 11.7%; *Blocked Discrimination*), 95.5% (*SD* = 9.1%; *Intermixed Discrimination*), 93.2% (*SD* = 10.6%; Triplet Discrimination), and 92.8% (*SD* = 10.3%; Identification) correct categorization responses, respectively, in the final two 10-trial blocks of the training stage. Final training performance did not differ significantly between groups, *F*(3,67) = 0.25; *p* = .86.

**Fig 4:**
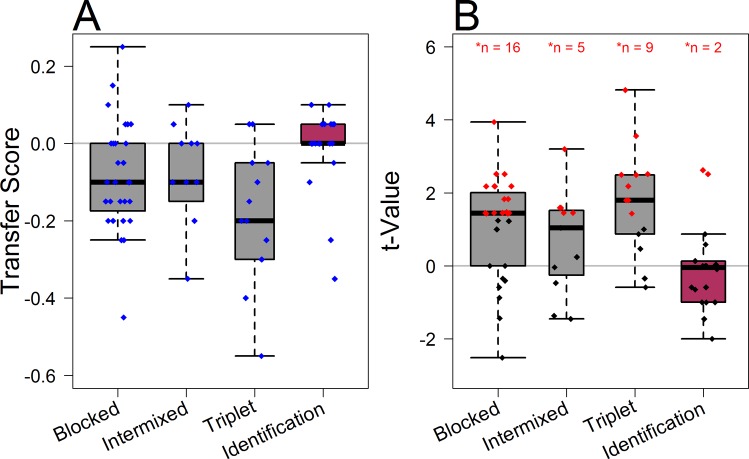
**A. Box plot of the transfer scores (performance in the last 20 trials of training subtracted from performance in the first 20 trials of transfer; note that a score of 0 corresponds to full transfer).** The thick black lines represent the medians, boxes indicate interquartile ranges, and whiskers refer to 1.5 interquartile ranges below and above the first and third quartile, respectively. The width of the boxes is proportional to the sample size in the respective groups. Individual transfer scores are depicted as small blue diamonds. **B. Individual *t*-statistics (low power) testing for a significant decrease in performance from the end of training to the initial transfer phase.** The thick black lines represent the medians, boxes indicate interquartile ranges, and whiskers refer to 1.5 interquartile ranges below and above the first and third quartile, respectively. The width of the boxes is proportional to the sample size in the respective groups. Individual *t*-values scores are depicted as small diamonds (significant *t*-values are printed in red). χ^2^ tests showed that the proportion of individuals showing significant specificity was considerably higher in groups Blocked, Intermixed, and Triplet, as compared to group Identification.

Given that all groups showed significant learning on their respective tasks, we then asked whether the learning transferred to the previously ‘unseen’ B/C transfer discriminations (note that all groups did categorize or identify both B and C feathers during training, but only the explicit B/C discriminations were omitted). As a measure of specificity of learning, we subtracted final training performance (i.e., mean accuracy in final two 10-trial blocks of training) from the initial transfer performance (i.e., mean accuracy in the first two 10-trial blocks of the transfer phase). In other words, in this metric, a score of zero would indicate full transfer (i.e., one would be performing the transfer task at the same level that one finished the training task), while negative numbers would indicate some degree of specificity (i.e., one would be performing the transfer task worse than one finished the training task). These learning-corrected transfer scores are illustrated in Fig 4A, and they differed significantly between groups, *F*(3,67) = 4.19; *p* = .009; η^2^ = 0.16 (note that this main effect was not contingent on our specific definition of the transfer score, group differences were found also with regard to alternative transfer scores covering the final and initial 30 trials of the training and transfer stages, F(3,67) = 3.91; p = .012; η^2^ = 0.15).

One-sided paired *t*-tests and Bayes factors (scaled to a Jeffrey-Zellner-Siow prior r = .707, 10000 iterations, contrasting a model with a phase difference in accuracy (H1) to a model without a phase difference (H0), using the R-package BayesFactor [54,55]) calculated within each group further confirmed that, on the group level, there was a significant decrease in accuracy from training to transfer after blocked, *t*(27) = 3.11; *p* = .002; *BF*_10_ = 9.33, intermixed, *t*(10) = 2.07; *p* = .03; *BF*_10_ = 1.40, and triplet category discrimination training, *t*(13) = 4.23; *p* < .001; *BF*_10_ = 43.12, indicating some degree of learning specificity. In contrast, there was no significant decrease in accuracy from final training to the initial transfer phase after category identification training, *t*(17) = 0.61; *p* = .27; *BF*_10_ = 0.29, suggesting that participants these participants successfully learned the rule to discriminate the crucial categories B and C during training and were thus able to apply their category knowledge to the subsequent B/C transfer discriminations task.

We then tested specificity for each individual by contrasting performance on all trials of the final two training blocks with performance on all trials of the initial two transfer blocks using *t*-tests (note that a significant difference suggests specificity, whereas non-significance may not necessarily indicate transfer trials were considered as independent measurement; to avoid a bias towards transfer we used a lax *p* < .1 criterion to categorize specificity). Fig 4B shows the resulting *t*-statistics for each participant in the four training conditions. It is evident that the proportion of participants who showed large *t*-values or significant specificity (red diamonds) was considerably lower in group Identification (2 of 18 participants) than in all the other groups (i.e., 9 of 14 in group Triplet, 5 of 11 in group Intermixed, and 16 of 28 in group Blocked). A 4×2 χ^2^ contingency-table test confirmed significant group differences in the relative frequency of transfer (as opposed to significant specificity), χ^2^(3) = 11.69; *p* = .009. Individual 2×2 χ^2^ tests revealed that the relative frequency of participants showing transfer in group Identification was significantly greater than in the other groups of participants who were trained on a Blocked, χ^2^(1) = 6.75; *p* = .009, Intermixed, χ^2^(1) = 4.46; *p* = .035, or Triplet Discrimination task, χ^2^(1) = 7.65; *p* = .006. All other group comparisons of the relative frequency of transfer (Blocked vs. Intermixed vs. Triplet) were not significant, χ^2^(1) < 1. We note that the above analyses assume a dichotomy that is based on an arbitrary cut-offs and the results should thus be used primarily for descriptive purposes. However, very similar results are obtained with other statistical approaches as well. For instance, the distributions of transfer scores (Fig 4A) in the three discrimination groups (Blocked, Intermixed, Triplet) were better fit by a unimodal distribution than a bimodal distribution (log likelihood deviances: *D*(3) = 1.95, *D*(3) = 1.50, *D*(3) = 0.44, respectively; all with means of the transfer score distribution that were less than zero, indicating some degree of learning specificity). The distribution of transfer scores in the Identification group meanwhile was better fit by a bimodal distribution (*D*(3) = 16.67). This distribution includes (as in clear in Fig 4A) a large cluster of individuals with transfer scores distributed around zero and a much smaller cluster of individuals with a transfer scores below zero. Thus, if we were to utilize this approach to categorize participants, all of the participants from the three discrimination groups would be categorized as belonging to a “not full transfer” group, while all but two of the participants in group Identification would be categorized as belonging to a “full transfer” group (which would, in turn, produce even more significant results than those utilizing the cut-off based approach above).

## Discussion

Predicting when training will produce enhancements that generalize across tasks is one of the central problems in the study of human learning. In the perceptual domain, it has been shown that the degree of observed learning specificity can be predicted by assessing the extent to which a given training paradigm supports a task- and/or stimulus-specific solution (along with the ease with which this specific solution can be learned; [10,26,27]). Generalization is observed in those cases where the training task is designed to eliminate learning strategies that are task- and/or stimulus-specific (or at least make such strategies extremely difficult).

Here we applied this same line of logic to rule-based category learning. Individuals trained on tasks that permitted a stimulus-specific solution (i.e., a series of rules separating certain categories), did not show immediate transfer to a previously untrained discrimination problem (i.e., they showed significant specificity). Conversely, those individuals trained on a task that did not easily permit learning of simple rules to separate specific categories showed immediate generalization of learning to the untrained discrimination problem. Because participants in all cases had roughly equivalent perceptual experience (i.e., had seen roughly the same stimuli), these results cannot be attributed to differences in the opportunity to acquire more general abilities. Instead, the results strongly point to the critical role of the task in determining what is learned during training, which then in turn predicts what new tasks can be performed given this learning.

Interestingly, significant specificity was observed even in some of the conditions where one might have expected some degree of transfer to occur [51–53]. For example, intermixing discriminations did not appear to result in significantly less specificity than having participants learn the rules in discrete blocks. Given the relatively small sample size though this negative result should be interpreted with caution.

That the discrimination tasks and the identification task fostered different category representations is consistent with the broader categorization literature [28,32,47,56,57]. Discrimination tasks encourage participants to selectively attend to discriminating features [58], while inference tasks encourage encoding the internal structure of the categories [41]. Another way to cast this pattern of results is that participants attempt simpler solutions before more complex ones [59]. For example, when presented with multi-dimensional stimuli with clear family-resemblance structure, participants tend to use only a single dimension to sort them into categories [60]. A similar bias towards adopting a unidimensional rule is also observed when participants are trained with delayed, indirect feedback [61].

Tasks that require participants to learn about the internal structure of the categories from which stimuli are sampled support generalization to new discrimination tasks involving either novel stimuli or untrained pair-wise distinctions [47,56]. This effect has previously been attributed to the fact that feature-inference requires tracking the covariance among the features and the probability of each feature given the presence or absence of others, while discrimination is often possible based on a subset of discriminating features. This cannot account for the effect in our study, however, because only a single feature dimension was diagnostic among the stimuli in our tasks and thus there is no covariance structure to track. These results are important because they show that different tasks require attending to a single stimulus dimension in different ways, which then leads to different representations being learned–despite the descriptions feature-inference and category-discrimination being formally identical [62]. Indeed, even manipulations as subtle as providing a category label simultaneously with each item versus having the participant guess the label before being provided the correct label cause different category representations to be learned [57].

In the categorization literature, a major distinction is made between tasks that allow for rule-based learning and those that require information integration (see [29] for a review). None of our tasks require information integration, because all tasks involved unidimensional discrimination, and so all should support rule-based learning. When we refer to learning a category boundary, this is in effect a rule. One clear future direction would be to examine transfer to previously unobserved stimuli where any of these rules could be directly applied to assess the extent to which the rules were global in nature, versus local within the space. That said, the Competition between Verbal and Implicit Systems (COVIS) model [37] and other similar models that put rule learning and maintenance under executive control predict that the explicit rule learning system should be taxed by learning multiple rules at once, and the data seem to support this prediction, which in turn might make learning four distinct categories (as employed here) extremely difficult for participants [63,64].

Critically, our transfer task could not be accomplished by applying a previously learned discrimination rule. For the training to transfer, participants need to learn the structure and extent of the categories themselves. All participants were provided with the same information, and it is enough to define the B and C categories. Yet only participants who were asked to identify category exemplars rather than discriminate them actually formed representations of categories B and C that were useful on the transfer task. So despite the task being a unidimensional problem, the participants trained to identify exemplars must have relied on some other learning mechanism.

It is interesting to juxtapose the discussion of learning to perform simple unidimensional discriminations in the categorization and perceptual learning literatures. In the categorization literature, unidimensional discriminations are considered to be easy to verbalize and thus amenable to an explicit rule-based encoding mediated by the prefrontal lobe, anterior cingulate, and head of the caudate nucleus [37]. On the other hand, perceptual learning of such discriminations are thought to be mediated by tuning of very low level visual cortical areas—to the point where shifting the stimulus presentation location within the visual field can negatively affect performance. While this may seem incongruous, when one considers that perceptual learning tasks are designed to press the limits of human acuity, it becomes apparent that a verbal rule may not have the appropriate fidelity to be useful in a perceptual learning task (indeed, in perceptual learning participants are nearly always explicitly given the categorization rule–e.g., “shifted left/shifted right”; “rotated clockwise/rotated counterclockwise”, etc.). This highlights the fact that the relation between task demands, learning mechanism, and potential for transfer are complex.

One point worth noting is that although we have described the learning that occurred in the identification group as being reflective of a “band” (essentially two independent rules separating members of a category from stimuli that are not members of the category; cf. [65]), the transfer that is observed is also consistent with learning something even richer. Namely, it is possible that the participants in the identification group learned the generative model for categories (i.e., the likelihood of a category given the features of a particular stimulus; cf. [66]). In practice, it can be quite difficult to determine experimentally if participants have learned a generative model (at least in tasks such as in this work where participants cannot easily generate their own data). However, this is an important distinction to be made in future research, perhaps using a task that allows for a clear method of separating whether an individual has learned a generative-model versus a more complex model-free solution (e.g., ‘bands’).

## Supporting Information

S1 Data The file CategoryTransfer_RawData.Rdata contains the raw data necessary to reproduce the results reported in this article. The variables are explained in a separate ReadMe.txt file.
(ZIP)
